# The molecular mechanism of MiR-26a-5p regulates autophagy and activates NLRP3 inflammasome to mediate cardiomyocyte hypertrophy

**DOI:** 10.1186/s12872-023-03695-w

**Published:** 2024-01-03

**Authors:** Li-qun Tang, Wei Wang, Qi-feng Tang, Ling-ling Wang

**Affiliations:** 1 Geriatric Medicine Center, Department of Geriatric Medicine, Zhejiang Provincial People ’ s Hospital, Affiliated People’s Hospital, Hangzhou Medical College, Hangzhou, Zhejiang China; 2 Department of Pharmacy, Zhejiang Province People’s Hospital, Hangzhou Medical College, No.156 Shangtang Road, Xiacheng District, Hangzhou, 310016 Zhejiang China; 3 Department of Radiology, Zhejiang Province People’s Hospital, Hangzhou, 310016 Zhejiang China; 4 Department of Critical Care Medicine, Dinghai District Central Hospital, Zhoushan, 316000 Zhejiang China

**Keywords:** miR-26a-5p, Cardiac hypertrophy, Apoptosis, Autophagy, Cardiomyocytes

## Abstract

**Objective:**

Many studies have found that miR-26a-5p plays an essential role in the progression of pathological cardiac hypertrophy, however, there is still no evidence on whether miR-26a-5p is related to the activation of autophagy and NLRP3 inflammasome. And the mechanism of miR-26a-5p and NLRP3 inflammasome aggravating pathological cardiac hypertrophy remain unclear.

**Methods:**

Cardiomyocytes were treated with 200µM PE to induce cardiac hypertrophy and intervened with 10mM NLRP3 inhibitor INF39. In addition, we also used the MiR-26a-5p mimic and inhibitor to transfect PE-induced cardiac hypertrophy. RT-qPCR and western blotting were used to detect the expressions of miR-26a-5p, NLRP3, ASC and Caspase-1 in each group, and we used α-SMA immunofluorescence to detect the change of cardiomyocyte area. The expression levels of autophagy proteins LC3, beclin-1 and p62 were detected by western blotting. Finally, we induced the SD rat cardiac hypertrophy model through aortic constriction (TAC) surgery. In the experimental group, rats were intervened with MiR-26a-5p mimic, MiR-26a-5p inhibitor, autophagy inhibitor 3-MA, and autophagy activator Rapamycin.

**Results:**

In cell experiments, we observed that the expression of miR-26a-5p was associated with cardiomyocyte hypertrophy and increased surface area. Furthermore, miR-26a-5p facilitated autophagy and activated the NLRP3 inflammasome pathway, which caused changes in the expression of genes and proteins including LC3, beclin-1, p62, ACS, NLRP3, and Caspase-1. We discovered similar outcomes in the TAC rat model, where miR-26a-5p expression corresponded with cardiomyocyte enlargement and fibrosis in the cardiac interstitial and perivascular regions. In conclusion, miR-26a-5p has the potential to regulate autophagy and activate the NLRP3 inflammasome, contributing to the development of cardiomyocyte hypertrophy.

**Conclusion:**

Our study found a relationship between the expression of miR-26a-5p and cardiomyocyte hypertrophy. The mechanism behind this relationship appears to involve the activation of the NLRP3 inflammasome pathway, which is caused by miR-26a-5p promoting autophagy. Targeting the expression of miR-26a-5p, as well as inhibiting the activation of autophagy and the NLRP3 inflammasome pathway, could offer additional treatments for pathological cardiac hypertrophy.

**Supplementary Information:**

The online version contains supplementary material available at 10.1186/s12872-023-03695-w.

## Introduction

Pathological myocardial hypertrophy is an independent risk factor for cardiovascular diseases. The continuous development of myocardial hypertrophy will cause myocardial ischemia, decreased myocardial compliance, arrhythmia, etc., eventually leading to severe consequences such as heart failure and sudden death [1, 2]. Therefore, the prevention and treatment of pathological myocardial hypertrophy have always been important in medical research. Pathological cardiac hypertrophy is often accompanied by pathological changes such as the increased size of cardiomyocytes, increased number of sarcomeres, increased protein synthesis, a proliferation of interstitial cells, and hyperplasia of fibrous tissue [3–6]. Although some genes and signalling pathways were involved in the pathological process of cardiac hypertrophy, their mechanism has yet to be fully elucidated, and further research is needed [7–10].

Relevant studies have found that interleukin-lβ (IL-1β) plays a vital role in the pathogenesis of cardiac hypertrophy. For example, IL-1β expression increased in cultured primary cardiomyocytes and animal experiments. And the increase of IL-1β expression is more evident in heart failure [11, 12], in addition, transgenic mice overexpressing IL-1β had cardiomyocyte hypertrophy and increased expression of ANP and β-MHC [13]. However, the splicing and maturation of IL-1β precursor require the participation of NLRP3 inflammasome [14]. In the previous study of our research group [15], we found that knocking down miR-26a-5p can improve pathological cardiac hypertrophy by regulating autophagy, but whether miR-26a-5p regulates autophagy is associated with NLRP3 inflammasome, which leads to the aggravation of pathological cardiac hypertrophy, and the regulatory mechanism between them need further study.

Therefore, our study intends to explore the expression and role of the NLRP3 inflammasome signalling pathway in pathological cardiac hypertrophy by constructing cell and animal models of pathological cardiac hypertrophy; Then, the regulation of miR-26a-5p on the NLRP3 inflammasome signalling pathway was explored. Finally, the molecular mechanism of miR-26a-5p regulating autophagy leading to NLRP3 inflammasome activation was further explored, and the potential clinical value of miR-26a-5p molecule as a diagnostic and therapeutic target for cardiac hypertrophy was elucidated.

## Materials and methods

### Cell culture and treatments

Rat cardiomyocytes H9C2 were purchased from Sebachem Biologicals (Shanghai, China). Cardiomyocytes were cultured in DMEM medium containing 10% fetal bovine serum, and the incubator environment was 37 °C, including 5% carbon dioxide and saturated humidity. The medium was changed and subcultured regularly. After a stable cell state, cardiomyocyte hypertrophy was induced with PE (200 µM).

### RNA extraction and real-time quantitative polymerase chain reaction (RT-qPCR)

According to the experimental grouping, after extracting total RNA from cardiomyocytes, the purity and concentration of RNA were determined, and the RNA was reverse transcribed into cDNA with Revertaid First Strand cDNA Synthesis Kit (THERMO). Then cDNA, primers and DEPC water were added in a 20ul system, and the SsoAdvance Universal SYBR Green SuperMix (BIO-RAD) system was used for automatic sample amplification. The results were calculated using the quantitative PCR analysis software BIO-RAD CFX Manager 3.1. Finally, using the target gene/GAPDH, the relative expression level of the target gene was determined by the 2^−ΔΔCt^ method. The primers of genes were as follows (Table 1).

**Table 1:** The primers of target genes

Name	Sequence(5’-3’)
GAPDH	CTCTCTGCTCCTCCCTGTTC
	TACGGCCAAATCCGTTCACA
NLRP3	CTGCATGCCGTATCTGGTTG
	ATGTCCTGAGCCATGGAAGC
ASC	ACAGTACCAGGCAGTTCGTG
	GGTCTGTCACCAAGTAGGGC
CASP1	CACGAGACCTGTGCGATCAT
	GCGCCACCTTCTTTGTTCAG
IL-1β	TTGAGTCTGCACAGTTCCCC
	GTCCTGGGGAAGGCATTAGG
U6	TTCGGCAGCACATATACTAAAGTTG
	GCGTGCCAGCCATCCTT
Rno-miR-26a-5p	GTCGTATCCAGTGCAGGGTCCGAGGTATTCGCACTGGATACGACAGCCTA
	CGCGTTCAAGTAATCCAGGA

### Western blotting analysis

The treated cells were added with appropriate RIPA lysis buffer (Beyotime Biotech, Shanghai, China) to extract total protein. Quantification and sample preparation were performed after complete protein extraction. After loading, the protein was separated by SDS-PAGE, transferred to the PVDF membrane, and blocked with 5% skimmed milk. Next, incubated overnight at 4 °C with the primary antibody and then incubated with the secondary antibody for 1 h at room temperature. After the PVDF membrane was washed for 30 min, the protein was developed with enhanced ECL reagent, and results were detected with a gel imaging system. Primary antibody concentrations were: LC3 (1/1000, Sangon Biotech Co., Ltd., Shanghai, China), beclin-1 (1/1000, Sangon Biotech Co., Ltd., Shanghai, China), P62 (1/1000, Sangon Biotech Co., Ltd., Shanghai, China), GAPDH (1/5000, Atagenix, Wuhan, China). And secondary antibody: goat anti-rabbit secondary antibody (1/5000). NLRP3 (1/1000, Shanghai Sangong Biotechnology Co., Ltd., China), ASC (1/1000, Shanghai Sangong Biotechnology Co., Ltd., China), Caspase-l (1/1000, Shanghai Sangong Biotechnology Co., Ltd., China). Secondary antibody concentration: goat anti-rabbit secondary antibody (1/5000). GAPDH was used as an internal reference, and the relative protein expression was calculated using the ImageJ system to read the grey values.

### Immunofluorescence and confocal microscopic assay

The cardiomyocytes were stained with α-SMA immunofluorescent after cell slides were made, and the image area changes were observed under a confocal fluorescence microscope. The detailed operation has been illustrated in our previous study [15].

### Animals and treatments

A total of 45 healthy SD rats were purchased from Hangzhou Medical College (Hangzhou, China), weighing 250 ± 20 g. NLRP3 inhibitor INF39 (cat.no. HY-101,868), autophagy inhibitor 3-MA (cat.no. HY-19,312), and autophagy activator Rapamycin (cat.no. HY-10,219) were obtained from MedChemExpress (Shanghai, China). miR-26a-5p mimic, miR-26a-5p mimic NC, miR-26a-5p inhibitor and miR-26a-5p inhibitor NC were synthesized by GenePharma (Shanghai, China). SD rats were randomly divided into sham operation group, aortic constriction (TAC) operation group, model (TAC) + INF39 treatment group, model (TAC) + miR-26a-5p mimic NC group, model (TAC) + miR-26a-5p mimic group, model (TAC) + miR-26a-5p mimic + autophagy Inhibitor 3-MA treatment group, model (TAC) + miR-26a-5p inhibitor NC group, model (TAC) + miR-26a-5p inhibitor group, model (TAC) + miR-26a-5p inhibitor + autophagy activator Rapamycin group, 5 rats in each group. In the TAC operation group, the model of pathological cardiac hypertrophy was established by transverse TAC operation. SD rats were intraperitoneally injected with a mixed anaesthetic of ketamine (8 mg/100 g), xylazine (2 mg/100 g) and atropine (0.06 mg/100 g), and then intubated tube connected ventilator to control breathing, tidal volume is 2-3 ml and respiratory rate is 90–110 times/min. The surgical area was cleaned, and a median anterior chest incision was made to open thoracotomy to the second rib, separated thymus exposed aortic arch. selected the needle pad hole according to the diameter of the aortic arch (1.0-1.1 mm), and prick the needle with a 26-27G pad (0.4-0.6 mm in diameter). Used No. 5 silk thread to narrow the aorta at 0.3 cm after the branch of the right common carotid artery. When the ligature is tight, remove the pad and close the mediastinum layer by layer. After the spontaneous breathing of SD rats recovered, the tracheal intubation was removed and transferred rats to the cage for routine feeding. Aortic arch ligation was not performed in the sham operation group, and the rest of the procedure was the same as in the operation group. In the model (TAC) INF39 treatment group, NLRP3 inhibitor INF3 was injected intraperitoneally once every 2 days while the routine feeding was carried out, and the model group was injected with normal saline as a control, SD rats were sacrificed with spinal cord dislocation 4 weeks later. The gene and protein expressions of miR-26a-5p, NLRP3, ASC, pro-Caspase-1 and Caspase-1 were detected by q-PCR and Western blot. The primers of target genes are listed in Table 1. In addition, the heart tissues of the rats were observed by haematoxylin–eosin (HE) staining.

### Immunohistochemical analysis

Fresh heart tissue from mice was fixed in 4% paraformaldehyde for over 24 h. After dehydration, the paraffin-soaked heart tissue was embedded in an embedding machine. After the wax solidified, the trimmed wax blocks were placed on a paraffin microtome and sectioned at 4 μm, and then the paraffin sections were dewaxed. Then, nuclei were stained with hematoxylin for 5–10 min. Stain the cytoplasm with eosin for 1–3 min. Placed the dehydration sealing sheet, put the section into 95% ethanol I 5 min, 95% ethanol II 5 min, absolute ethanol I 5 min, absolute ethanol II 5 min, xylene I 5 min, xylene II 5 min dehydration, then use neutral gum seal. Finally, staining results were checked with an upright optical microscope (Olympus Corporation, Japan), followed by image acquisition and analysis.

### Statistical analysis

All data were statistically analyzed using GraphPad Prism software (Graph Pad Software, Inc., San Diego, California, USA) and expressed as mean ± standard deviation. The t-test was used to compare two groups, one-way ANOVA was used to compare multiple groups, and post-hoc two sample comparison tests using S-N-K, LSD. Data used in plotting are repeated at least 3 times, and results were considered statistically significant when a *P* value < 0.05.

## Results

### miR-26a-5p and pyroptosis-associated proteins were up-regulated in PE-induced cardiac hypertrophy

**Fig. 1:**
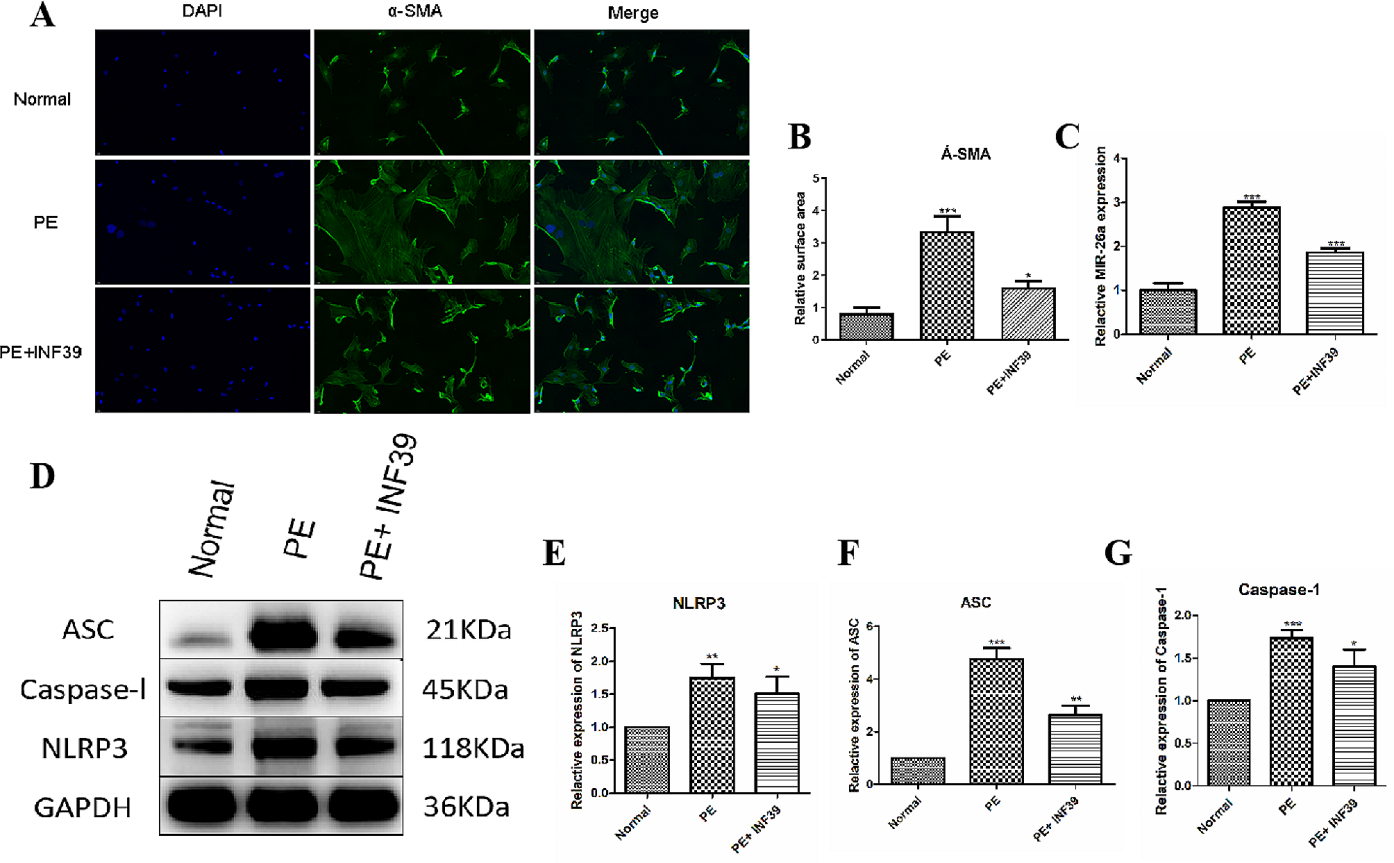
miR-26a-5p and pyroptosis-associated proteins were up-regulated in PE-induced cardiac hypertrophy. (**A**): Representative images of cardiac hypertrophy, as shown by a-SMA immunofluorescence. The nucleus was stained with DAPI (blue). The scale bar shows 10 μm. (**B**): Cell surface area was measured using anti-SMA staining (green) under fluorescence microscopy. (**C**): The mRNA expression of miR-26a-5p was detected in PE-induced cardiac hypertrophy by RT-qPCR, (**D**-**G**): The proteins expression of miR-26a-5p, ACS, NLRP3, Caspase-1 were detected in PE-induced cardiac hypertrophy by Western blotting. (*: *p*-value < 0.05; **: *p*-value < 0.01; ***: *p*-value < 0.001.)

Previously, 200 µM PE was used in our study to induce the hypertrophy model of cardiomyocytes. Immunofluorescence and confocal microscopy analysis revealed significantly higher expression of α-SMA in myocardial tissue of rats with PE-induced cardiac hypertrophy compared to the control group (Fig. 1A). After PE treatment, the cell surface area exhibited a significant increase (*p* < 0.001); however, after the intervention of INF39, a significant decrease was observed (*p* < 0.05), as demonstrated in Fig. 1B. Furthermore, we performed q-PCR and Western blotting to detect miR-26a-5p and pyroptosis-associated protein expression. Our findings revealed that the expression of miR-26a-5p was considerably up-regulated in the PE group (*p* < 0.001), whereas gene expression was significantly down-regulated following INF39 intervention (*p* < 0.05), as illustrated in Fig. 1C. Western blot analysis revealed a significant increase in the expression of miR-26a-5p, ACS, NLRP3, and Caspase-1 in the PE group (*p* < 0.001). Additionally, gene expression was significantly upregulated following INF39 intervention (*p* < 0.01) (Fig. 1D-G).

### miR-26a-5p activated autophagy leading to NLRP3 inflammasome activation in a PE-induced cardiomyocyte hypertrophy model

**Fig. 2:**
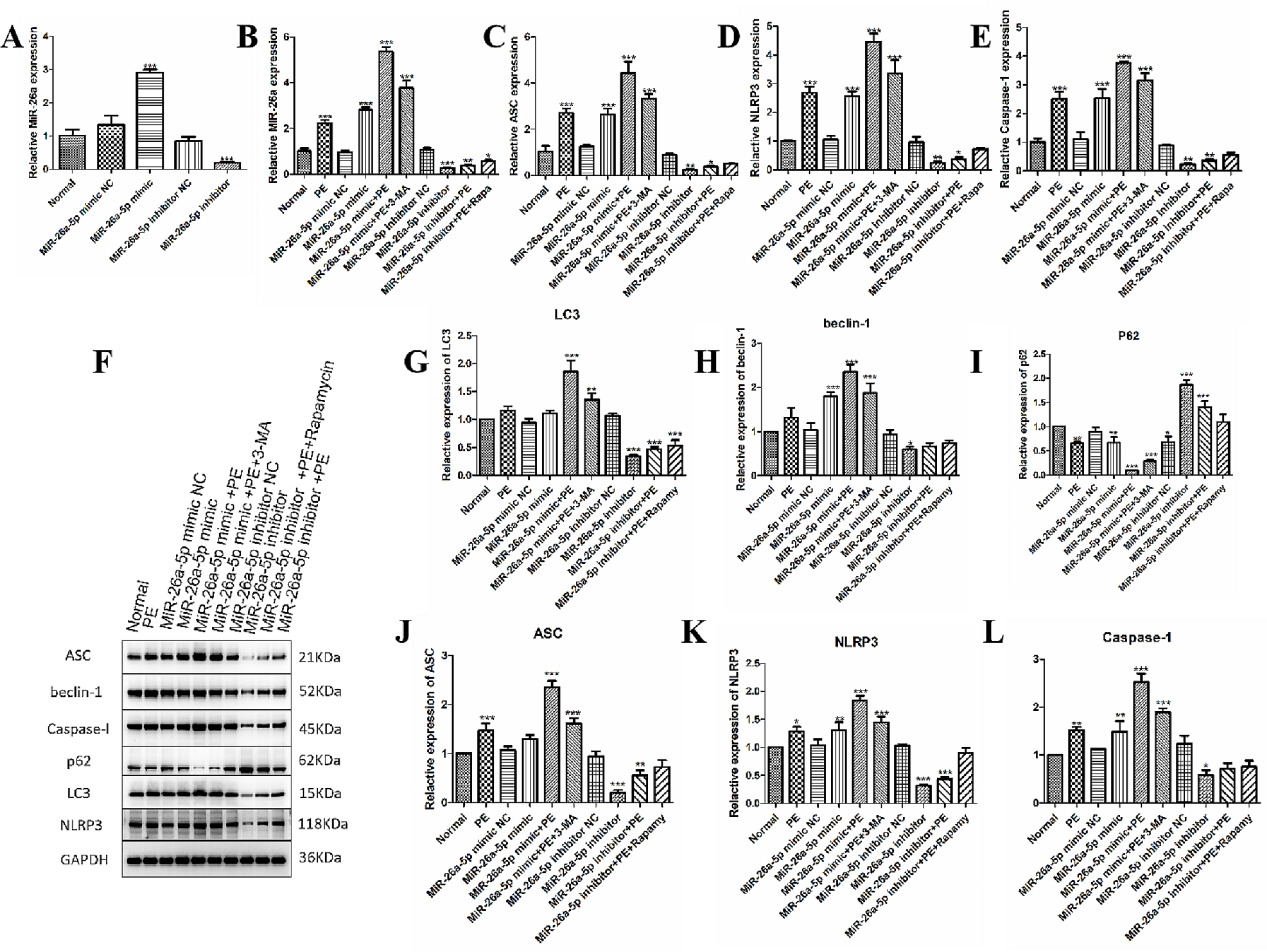
miR-26a-5p overactivated autophagy leading to NLRP3 inflammasome activation in a PE-induced cardiomyocyte hypertrophy model. (**A**): The mRNA expression of miR-26a-5p after mimic and inhibitor intervened cardiomyocytes. (**B**-**E**): The mRNA expression of miR-26a-5p, ACS, NLRP3, and Caspase-1 was detected in PE-induced cardiac hypertrophy by RT-qPCR. (**F**-**L**): Western blotting detected the protein expression of miR-26a-5p, LC3, beclin-1, p62, ACS, NLRP3, and Caspase-1 in PE-induced cardiac hypertrophy. (*: *p*-value < 0.05; **: *p*-value < 0.01; ***: *p*-value < 0.001.)

MiR-26a-5p was found to be up-regulated in cardiac hypertrophy induced by PE, potentially contributing to the hypertrophic processes. Therefore, we further explored the mechanism of miR-26a-5p-induced cardiomyocyte hypertrophy. Firstly, we detected the expression of miR-26a-5p after used miR-26a-5p mimic and inhibitor; q-PCR results showed (Fig. 2A) after miR-26a-5p mimic transfected cells, the expression of miR-26a-5p was significantly increased (*p* < 0.001), after used miR-26a-5p inhibitor, the expression of miR-26a-5p was inhibited considerably (*p* < 0.001). Furthermore, in the PE model, we observed that the expression of miR-26a-5p, ACS, NLRP3, and Caspase-1 was significantly higher than the control group (*p* < 0.001), after transfection with miR-26a-5p mimic, compared with a control group, the expression of miR-26a-5p, ACS, NLRP3, and Caspase-1 in miR-26a-5p mimic + PE group was more significantly increased (*p* < 0.001). Gene expression was down-regulated considerably after the autophagy inhibitor 3-MA treatment (*p* < 0.001). However, after miR-26a-5p inhibitor intervened cardiomyocytes, the expression of miR-26a-5p, ACS, NLRP3, and Caspase-1 was significantly inhibited compared with the control group (*P* < 0.01), and similar results were also found in miR-26a-5p inhibitor + PE group (*p* < 0.05). After treatment with autophagy activator Rapamycin, there was no significant difference between the miR-26a-5p inhibitor + PE + Rapa group and the control group; The results are shown in Fig. 2B-E.

We then examined the expression of autophagy- and pyroptosis-related. We observed that the expression levels of LC3 and beclin-1 were significantly increased (*p* < 0.001), and the expression levels of ACS, NLRP3 and caspase-1 were also increased (*p* < 0.01) compared to the control group. However, p62 was significantly decreased in the PE group (*p* < 0.01). After miR-26a-5p mimic transfected cardiomyocytes, the expression levels of LC3, beclin-1, ACS, NLRP3, and Caspase-1 showed a more significant increase (*p* < 0.001), while p62 exhibited a more significant decrease (*p* < 0.001). But after treatment with the autophagy inhibitor 3-MA, the expression levels of LC3, beclin-1, ACS, NLRP3, and Caspase-1 were significantly decreased (*p* < 0.001). In addition, p62 was significantly up-regulated (*p* < 0.001). We also observed that the expression levels of LC3, beclin-1, ACS, NLRP3, and Caspase-1 were significantly inhibited (*p* < 0.001), and p62 was significantly up-regulated (*p* < 0.001) after miR-26a-5p intervened cardiomyocytes. When we treated with the autophagy activator Rapamycin, the protein expression of LC3, beclin-1, p62, ACS, NLRP3, and Caspase-1 was slightly inhibited or showed no significant difference compared with the control group; The results are shown in Fig. 2F-L.

### Expression of miR-26a-5p was associated with PE-induced changes in the cardiomyocyte area

**Fig. 3:**
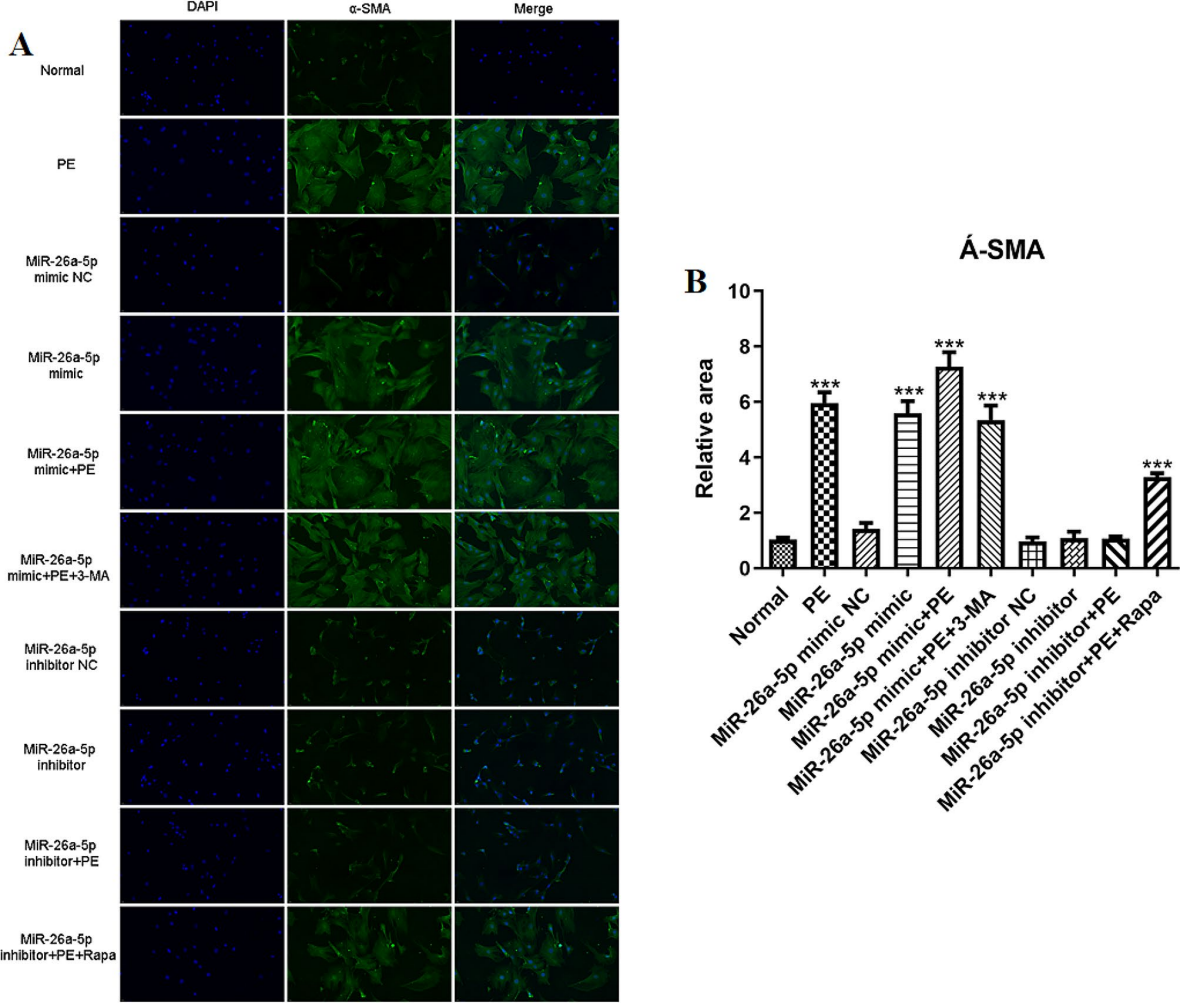
A-SMA immunofluorescence analysis of cardiomyocytes. (**A**): Representative images of cardiac hypertrophy intervened by miR-26a-5p mimic and inhibitor, as shown by a-SMA immunofluorescence. The nucleus was stained with DAPI (blue). The scale bar shows 10 μm. (**B**): Cell surface area was measured using anti-SMA staining (green) under fluorescence microscopy. (*: *p*-value < 0.05; **: *p*-value < 0.01; ***: *p*-value < 0.001.)

We then used Immunofluorescence and confocal microscopic assay to detect whether the expression of miR-26a-5p was related to the change of cardiomyocyte area induced by PE. We observed that PE significantly increased the cell surface area (*p* < 0.001), and the increase was more significant after transfection with miR-26a-5p mimic (*p* < 0.001). However, cell surface area decreased after intervention with the autophagy inhibitor 3-MA (*p* < 0.05). Conversely, the cell surface area was significantly down-regulated after intervention with miR-26a-5p inhibitor (*p* < 0.01), but the cell surface area was significantly increased after the intervention of autophagy activator Rapamycin (*p* < 0.01) (Fig. 3). This phenomenon shows that miR-26a-5p expression was associated with PE-induced changes in the cardiomyocyte area.

### Expression of miR-26a-5p leaded to cardiac structural changes in TAC model rats

**Fig. 4:**
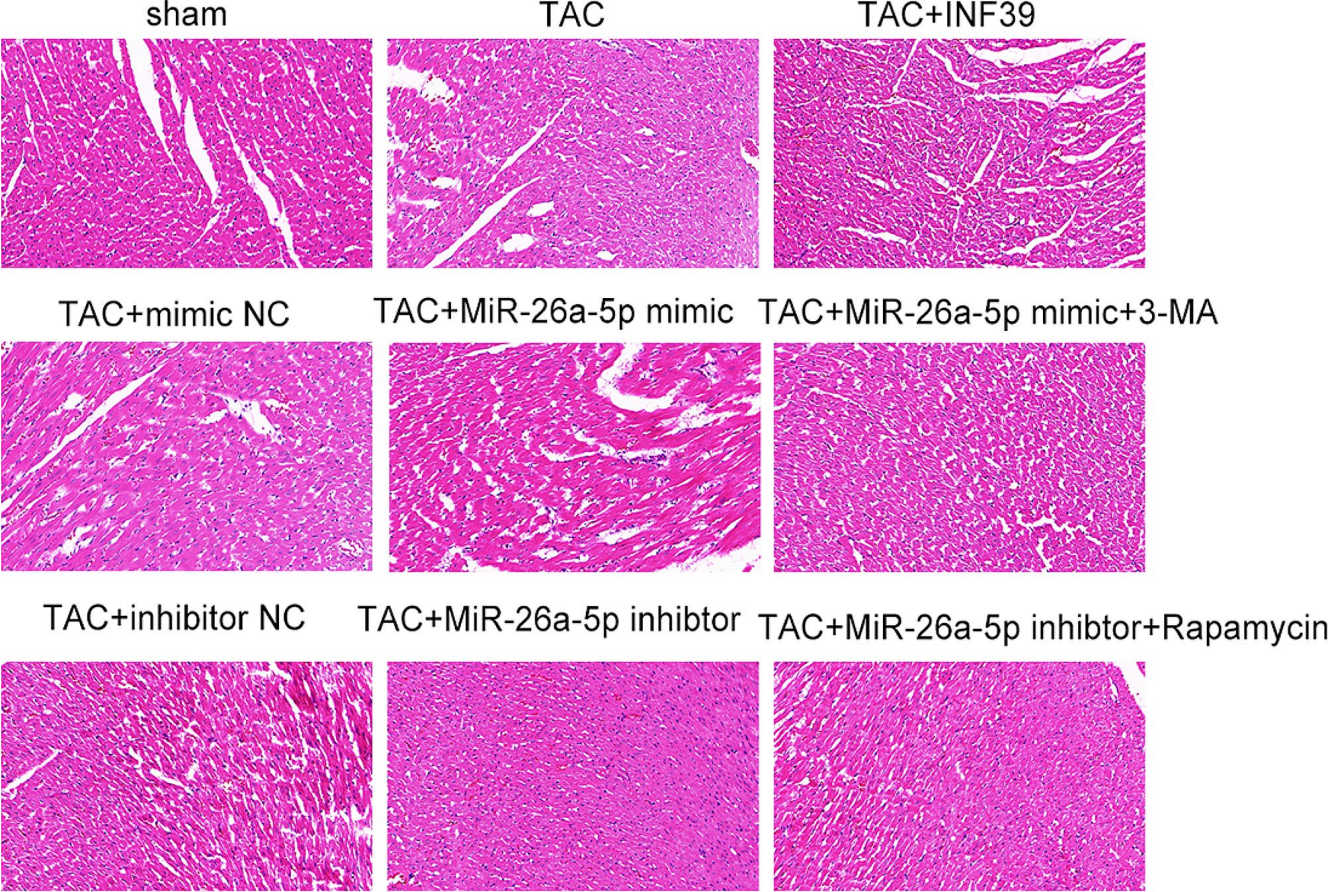
HE staining observation of heart tissue. (*: *p*-value < 0.05; **: *p*-value < 0.01; ***: *p*-value < 0.001.)

Our study investigated the structural changes in the heart of TAC model rats after intervention with miR-26a-5p mimic and miR-26a-5p inhibitor. HE staining showed that in the sham-operated group, the shape of the heart was standard, the cardiomyocytes were neatly arranged and the intercellular space was clear. Compared with the model (TAC) group, the model (TAC) + miR-26a-5p mimic + autophagy inhibitor 3-MA treatment group and the model (TAC) + miR-26a-5p inhibitor group had significantly improved cardiomyocyte enlargement and cardiac interstitial and perivascular fibrosis. In the other groups, the cardiomyocytes were disorganised and the cardiac fibres were visibly thickened and enlarged (Fig. 4). This phenomenon is evidence that miR-26a-5p is involved in the progression of pathological cardiac machine hypertrophy.

### miR-26a-5p was associated with NLRP3 inflammasome activation in TAC model rats

**Fig. 5:**
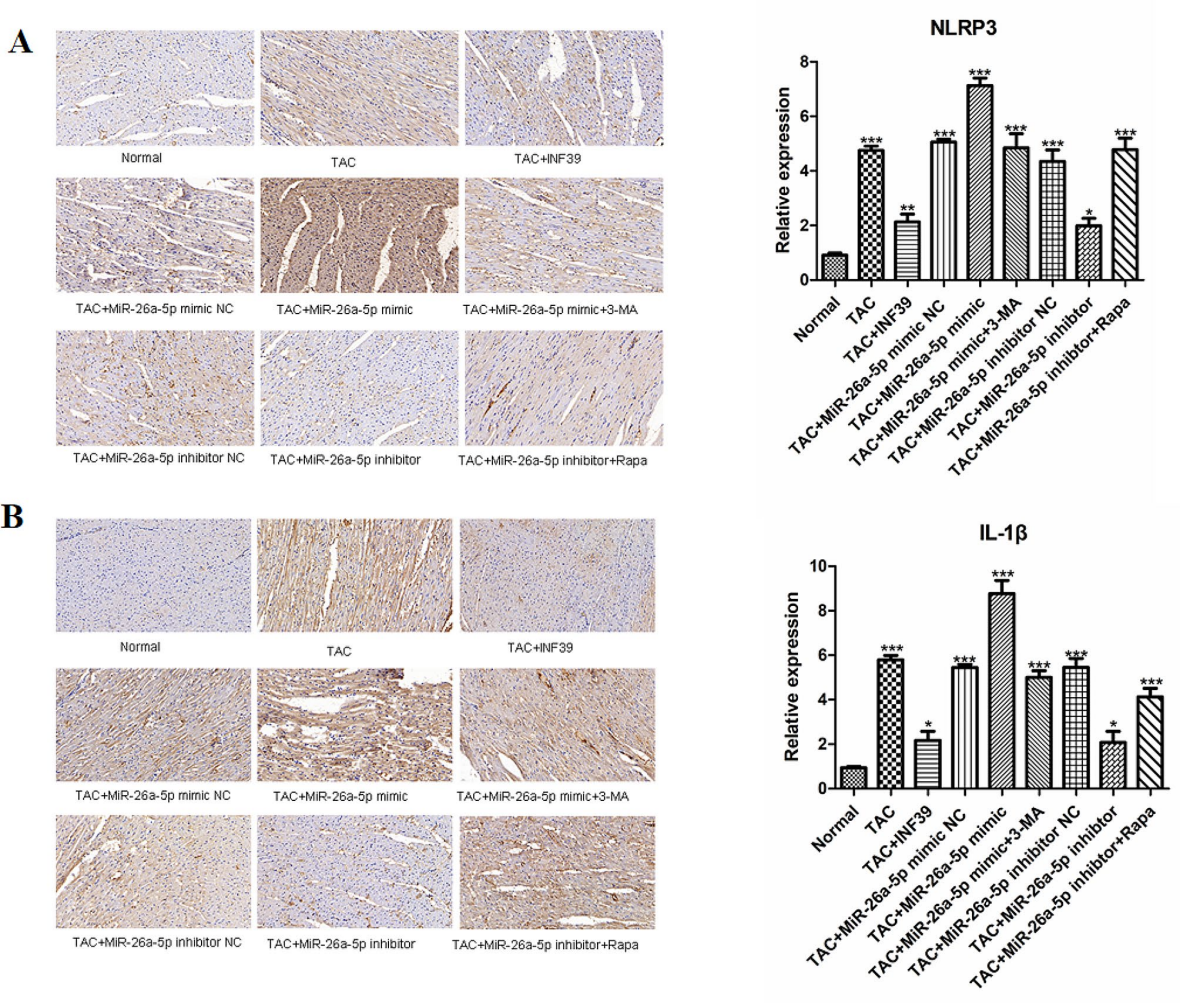
Immunohistochemical analysis NLRP3 and IL-β protein expression levels. (**A**): Immunohistochemical analysis NLRP3 and IL-β protein expression levels. (**B**): The relative protein expression levels of NLRP3 and IL-β. (*: *p*-value < 0.05; **: *p*-value < 0.01; ***: *p*-value < 0.001.)

First, we used immunohistochemical to analyse the specific mechanism of miR-26a-5p-induced cardiac hypertrophy in TAC rats. We observed that NLRP3 and IL-β expression was significantly upregulated (*p* < 0.001) in the TAC group compared with the control group, and significantly decreased after INF39 intervention. We also found that TAC miR-26a-5p mimic group expression was significant (*p* < 0.001). However, it was improved after treatment with the autophagy inhibitor 3-MA or intervention with the miR-26a-5p inhibitor (Fig. 5).

**Fig. 6:**
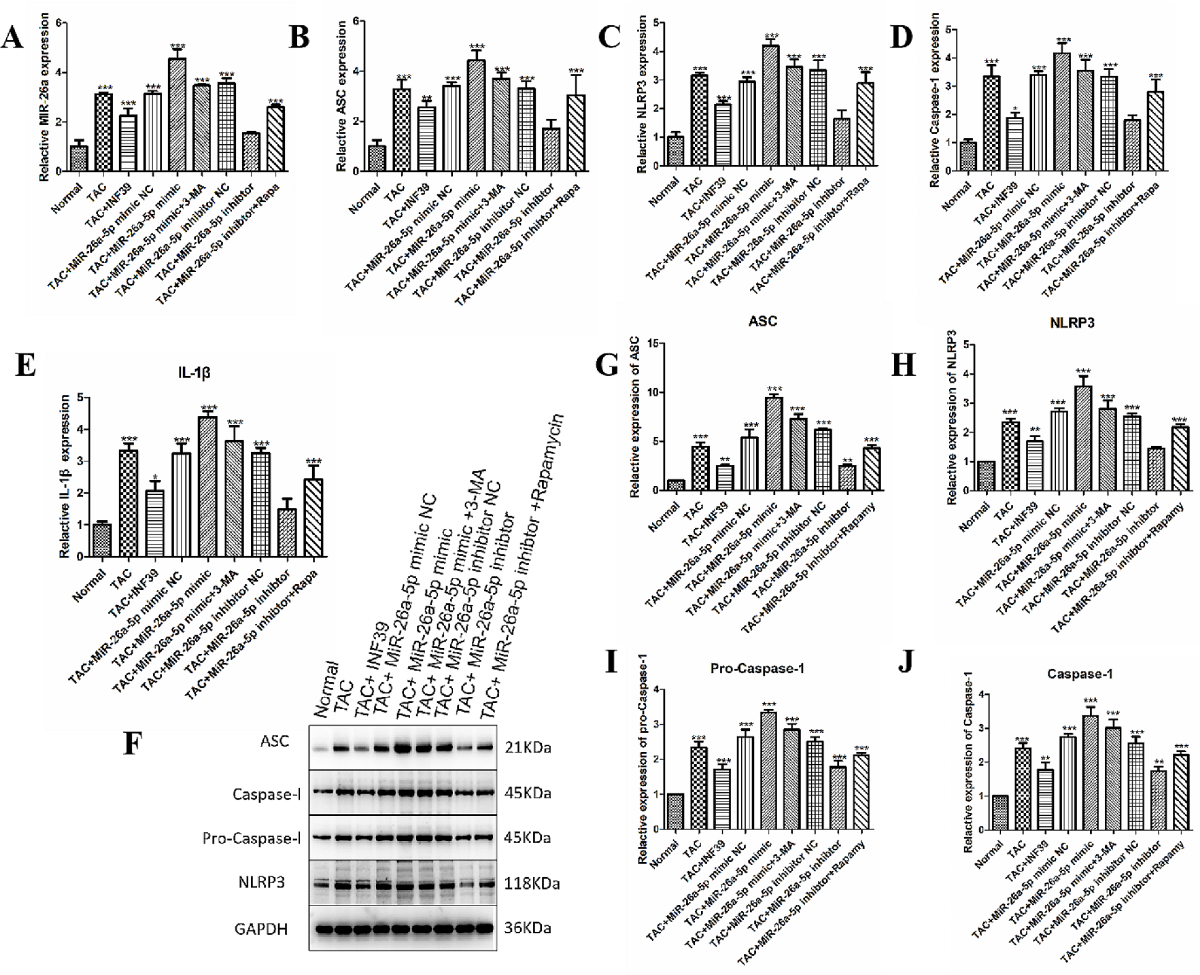
Expression levels of NLRP3 inflammasome pathway mRNA and proteins. (**A**-**E**): The mRNA expression of miR-26a-5p, ACS, NLRP3, Caspase-1 and IL-1-β. (**F**-**J**): The protein expression levels of ACS, NLRP3, pro-Caspase-1 and Caspase-1. (*: *p*-value < 0.05; **: *p*-value < 0.01; ***: *p*-value < 0.001.)

Rat heart tissue was extracted to detect NLRP3 and IL-β mRNA and protein expression levels. Q-PCR results (Fig. 6A-E) showed that compared to the control group, the TAC model group had significantly higher expression of miR-26a-5p, ACS, NLRP3, Caspase-1, and IL-1-β (*p* < 0.001). After miR-26a-5p mimic transfection of cardiomyocytes, the expression of miR-26a-5p, ACS, NLRP3, Caspase-1, and IL-1-β increased significantly in comparison to the control group (*p* < 0.001). Gene expression was significantly reduced following treatment with the autophagy inhibitor 3-MA (*p* < 0.001). However, there was no significant difference in the expression of miR-26a-5p, ACS, NLRP3, Caspase-1, and IL-1-β in the TAC + miR-26a-5p inhibitor group compared to the control group following miR-26a-5p inhibitor intervention (*P* > 0.05).

We also observed that compared with the control group, the TAC model group’s protein expression levels of ACS, NLRP3, pro-Caspase-1, and Caspase-1 increased (*p* < 0.001). Following transfection of cardiomyocytes with miR-26a-5p mimic, the expression levels of ACS, NLRP3, pro-Caspase-1 and Caspase-1 were even more significantly elevated (*p* < 0.001). However, the expression levels of ACS, NLRP3, pro-Caspase-1 and Caspase-1 were significantly decreased (*p* < 0.001) after treatment with the autophagy inhibitor 3-MA. Furthermore, it was noted that the expression of NLRP3 and pro-Caspase-1 was significantly suppressed (*p* < 0.001) following intervention with miR-26a-5p inhibitor, whereas there was no significant difference in the expression of ACS and Caspase-1, compared to the control group (*p* > 0.05). In addition, treatment with the autophagy activator Rapamycin effectively increased the protein expression of ACS and pro-Caspase-1 (*p* < 0.001), and NLRP3 also increased (*p* < 0.01); results were shown in Fig. 6F-J. Together, these data suggest that miR-26a-5p may activate autophagy leading to NLRP3 inflammasome activation in cardiomyocyte hypertrophy.

## Discussion

The NLRP3 inflammasome is one of the most extensively researched inflammasomes currently known, and is composed of NOD-like receptors, the adapter protein ASC and the effector protein Caspase-1 (IL-1β converting enzyme). This inflammasome can promote the splicing and maturation of IL-1β, IL-18 and IL-33 [16]. NLRP3 inflammasome activation can occur due to various exogenous or endogenous stress factors, such as infection, reactive oxygen species (ROS), damage, metabolites, and adenosine triphosphate (ATP) [17–20]. The aforementioned findings indicate that the NLRP3 inflammasome could be a crucial factor in the pathogenesis of cardiac hypertrophy. However, the precise role and underlying molecular mechanisms of NLRP3 inflammasome in cardiac hypertrophy remain ambiguous. Existing literature reports a close relation between autophagy and inflammasomes. Specifically, inflammasomes have the potential to trigger autophagy, while autophagy can modulate inflammasome activity [21–24].

Many studies have shown that autophagy can negatively or positively regulate the activation of NLRP3 inflammasome. At the same time, the NLRP3 inflammasome also reverses the effect of autophagy [25, 26]. For example, a study found that the acetylation of Atg5 can inhibit the maturation of autophagosomes [27], and SIRT3 can form a complex with Atg5 in cells to inhibit the acetylation of endogenous Atg5, thereby promoting the maturation of autophagosomes. However, the NLRP3 inflammasome was much more abundant in SIRT3-deficient cells than in normal cells. Therefore, there is a certain negative regulatory relationship between autophagy and NLRP3 inflammasome. Similarly, Chang et al. [28] resveratrol (RSV), a polyphenolic compound naturally produced in plants, can induce autophagy by activating the p38 gene, inhibit the activation of NLRP3 inflammasome in macrophages, and alleviate the body’s inflammatory response. Zhou et al. [29] found that berberine can inhibit the activation of NLRP3 inflammasome by up-regulating the autophagy level of macrophages to inhibit the inflammatory response. Reducing the level of beclin-1 in cells, or adding an autophagy inhibitor, could reverse the inhibition of berberine on NLRP3 inflammasome. However, Dupont et al. [30] found that autophagy can also positively regulate the activation of NLRP3 inflammasomes. In this study, autophagy under starvation conditions can enhance the activation of caspase 1 through an Atg5-dependent non-canonical pathway, promote the activation of inflammasomes, and increase IL-1β, IL-18 Synthesis of pro-inflammatory cytokines. At the same time, because some cytoplasmic proteins lack signal peptides, pro-inflammatory factors such as IL-1β and IL-18 are degraded, which instead promotes their excretion in the cytoplasm, further aggravating the inflammatory damage of tissue.

In summary, autophagy has a dual role in regulating inflammatory response, which depends on a certain cellular environment. Cellular autophagy can regulate inflammatory response [31], and autophagy can also be induced by cytokines [32, 33]. The specific mechanism remains to be further researched. The role of miR-26a-5p on cardiac hypertrophy has been elucidated in many studies [15, 34–36], and its mechanism may be related to the regulation of autophagy. Therefore, we constructed cell and animal cardiac hypertrophy models to explore whether miR-26a-5p could regulate autophagy, activate inflammasomes and lead to the aggravation of pathological cardiac hypertrophy.

In our research, we observed that the expression of miR-26a-5p is associated with cardiomyocyte hypertrophy and rat heart structure. Additionally, our results suggest that miR-26a-5p may activate autophagy, leading to the activation of the NLRP3 inflammasome and subsequently causing an increase in cardiac hypertrophy. Our research results have enhanced the understanding of the NLRP3 inflammasome signalling pathway on the pathological mechanism of cardiac hypertrophy; In addition, miR-26a-5p regulates autophagy and activates the NLRP3 inflammasome, thus mediating cardiomyocyte hypertrophy, These findings introduce novel insights for early diagnosis and drug development in the treatment of cardiac hypertrophy.

However, our research still needs to improve, and the specific mechanism of miR-26a-5p regulating the NLRP3 signalling pathway still needs further discussion. Because there is no evidence to suggest that over-expressing miR-26a-5p in an otherwise wildtype background can also cause hypertrophy, targeting the NLRP3 inflammasome pathway will benefit the treatment of myocardial hypertrophy is unknown, and whether miR-26a-5p can regulate the NLRP3 pathway to prevent myocardial hypertrophy needs more research to verify. In addition, our study did not further validate whether autophagy directly affects NLRP3 inflammatory vesicles, which requires further discussion. But overall, exploring the relationship between autophagy and NLRP3 inflammatory may provide new ideas for the treatment and prognosis of cardiac hypertrophy.

## Conclusion

Our study found a relationship between the expression of miR-26a-5p and cardiomyocyte hypertrophy. The mechanism behind this relationship appears to involve the activation of the NLRP3 inflammasome pathway, which is caused by miR-26a-5p promoting autophagy. Targeting the expression of miR-26a-5p, as well as inhibiting the activation of autophagy and the NLRP3 inflammasome pathway, could offer additional treatments for pathological cardiac hypertrophy.

### Electronic supplementary material

Below is the link to the electronic supplementary material.

Supplementary Material 1

## Data Availability

The datasets supporting the conclusions of this article are included within the article (and its additional files).
