# Supplementary material for: The molecular mechanism of MiR-26a-5p regulates autophagy and activates NLRP3 inflammasome to mediate cardiomyocyte hypertrophy
Source: BMC Cardiovasc Disord. 2024 Jan 3;24:18. doi: 10.1186/s12872-023-03695-w (PMC10765805; doi:10.1186/s12872-023-03695-w)
Supplement: Supplementary file 1 — Supplementary Material 1 [file 12872_2023_3695_MOESM1_ESM.docx]

**Figure 1D**

**Grouping from left to right: Normal, PE, PE+INF39**

**
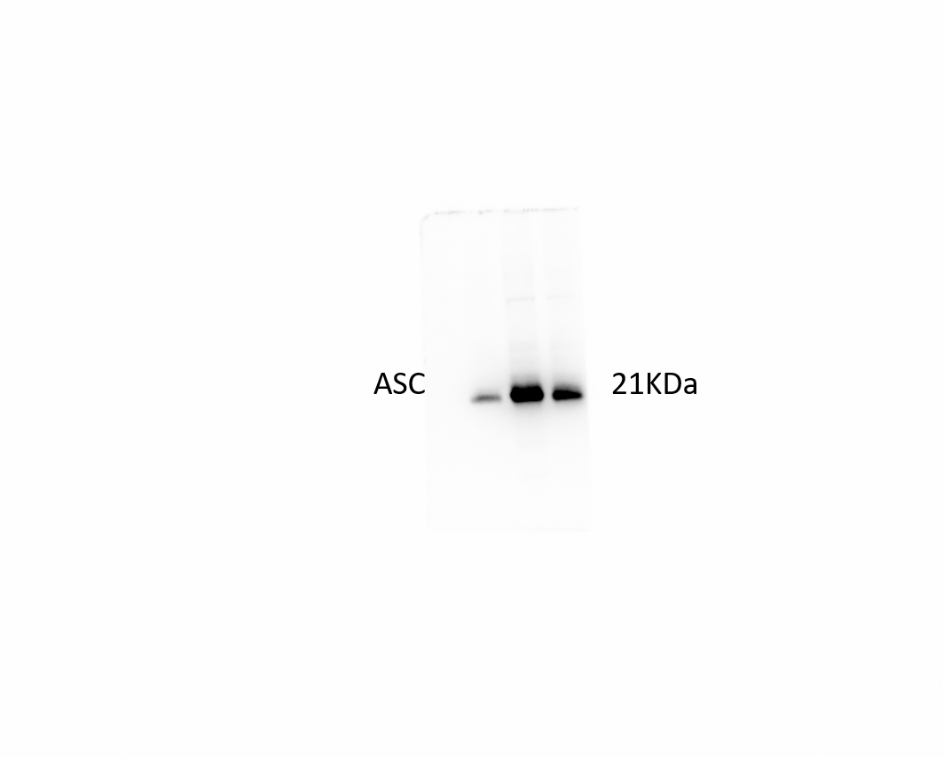
**

**
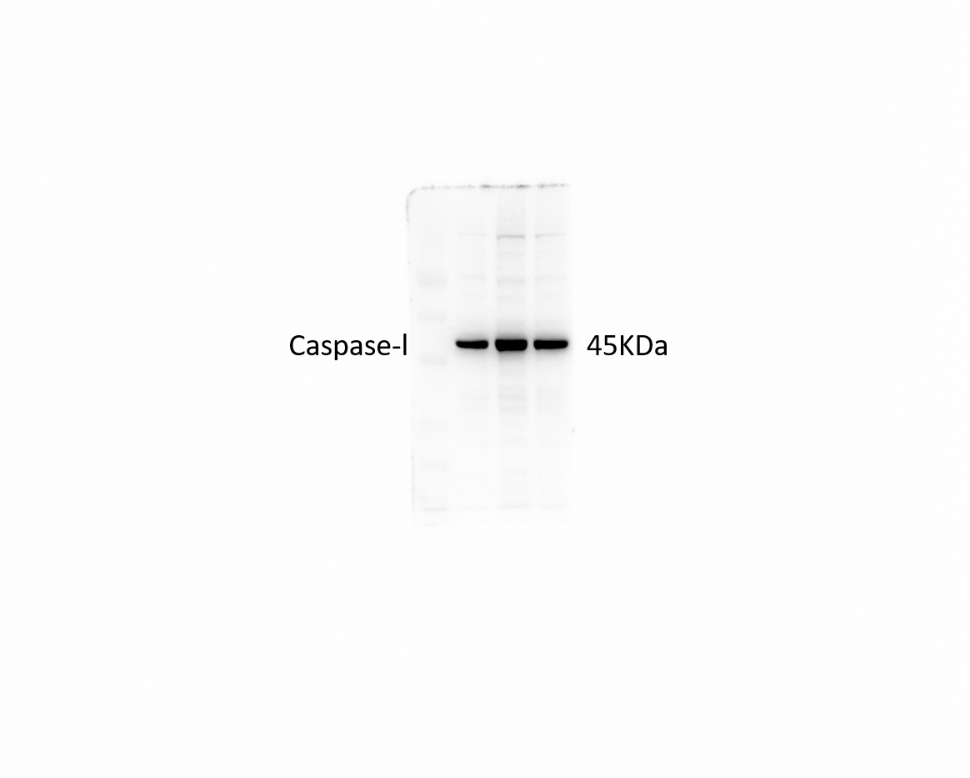
**

**
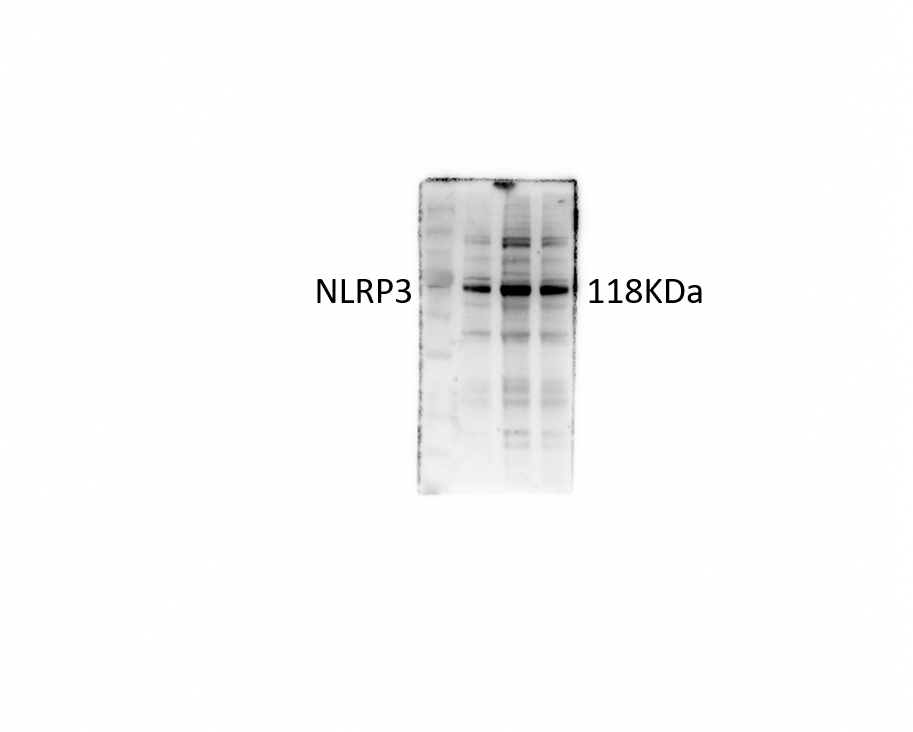
**

**
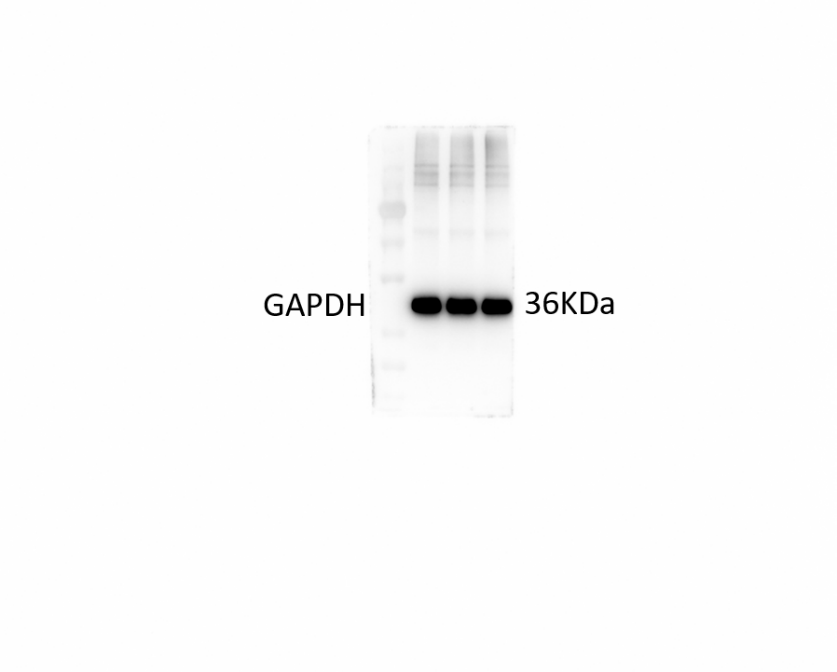
**

**Figure 2F**

**Grouping from left to right: Normal, PE, MiR-26a-5p mimic NC, MiR-26a-5p mimic, MiR-26a-5p mimic+PE, MiR-26a-5p mimic+PE+3-MA, MiR-26a-5p inhibitor NC, MiR-26a-5p inhibitor, MiR-26a-5p inhibitor+PE+Rapamy, MiR-26a-5p inhibitor+PE**

**
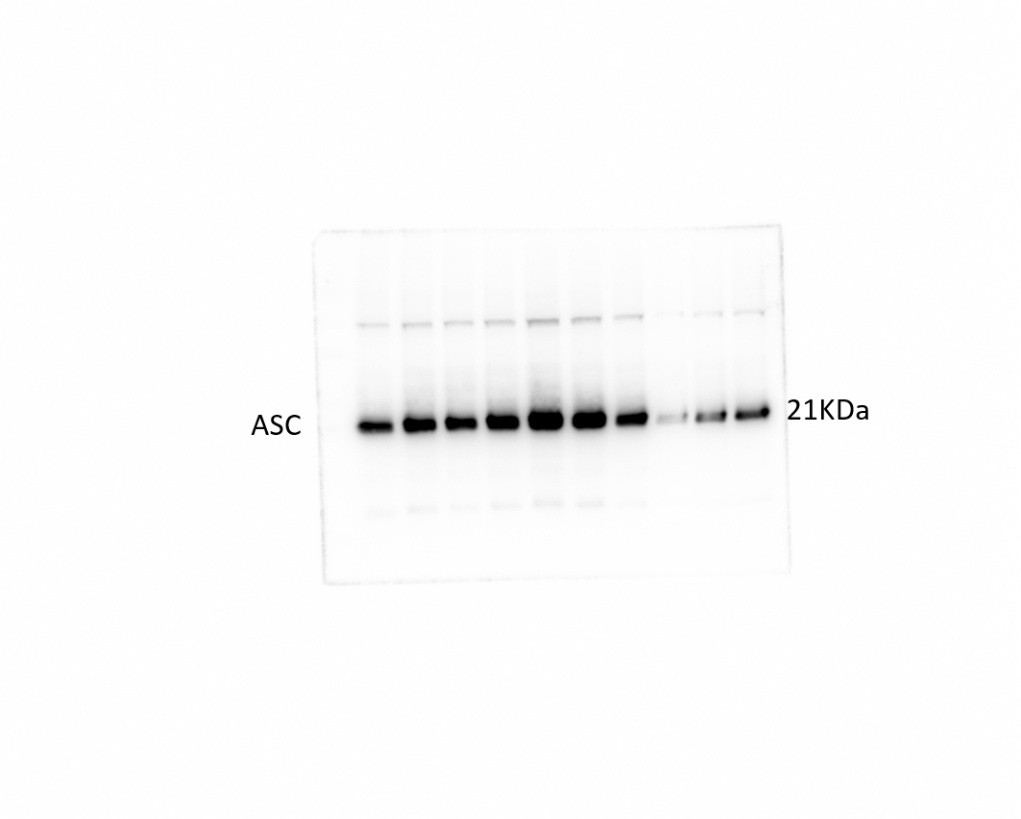

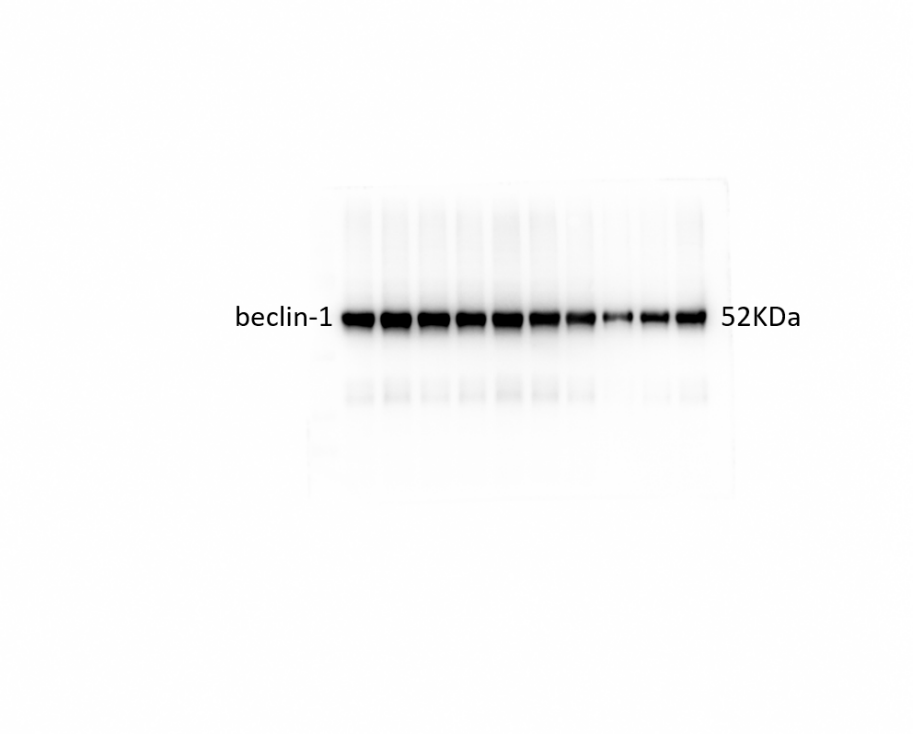
**

**
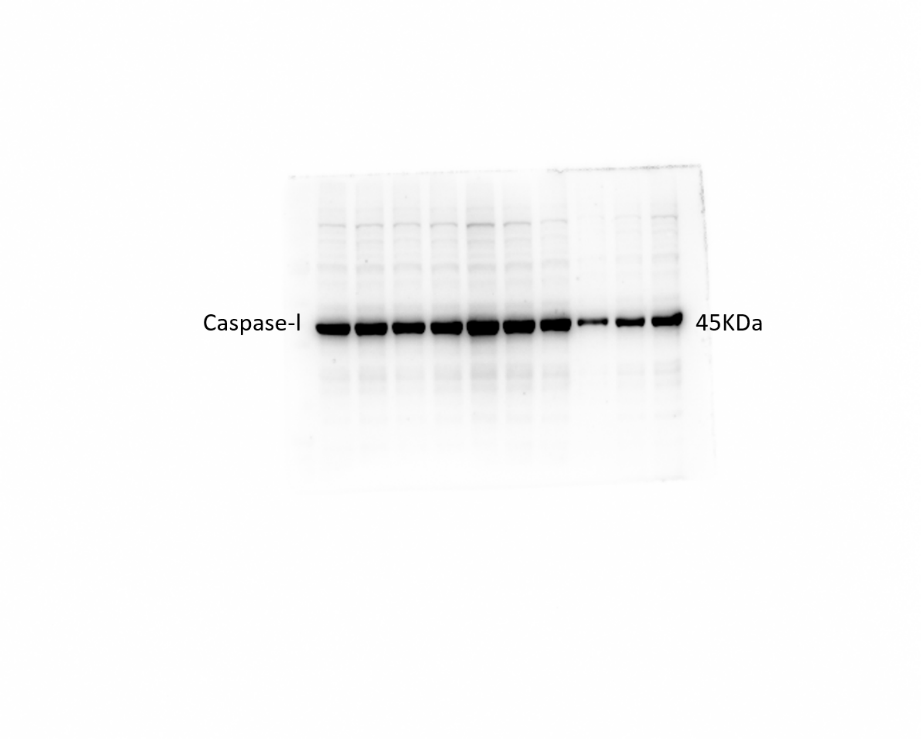

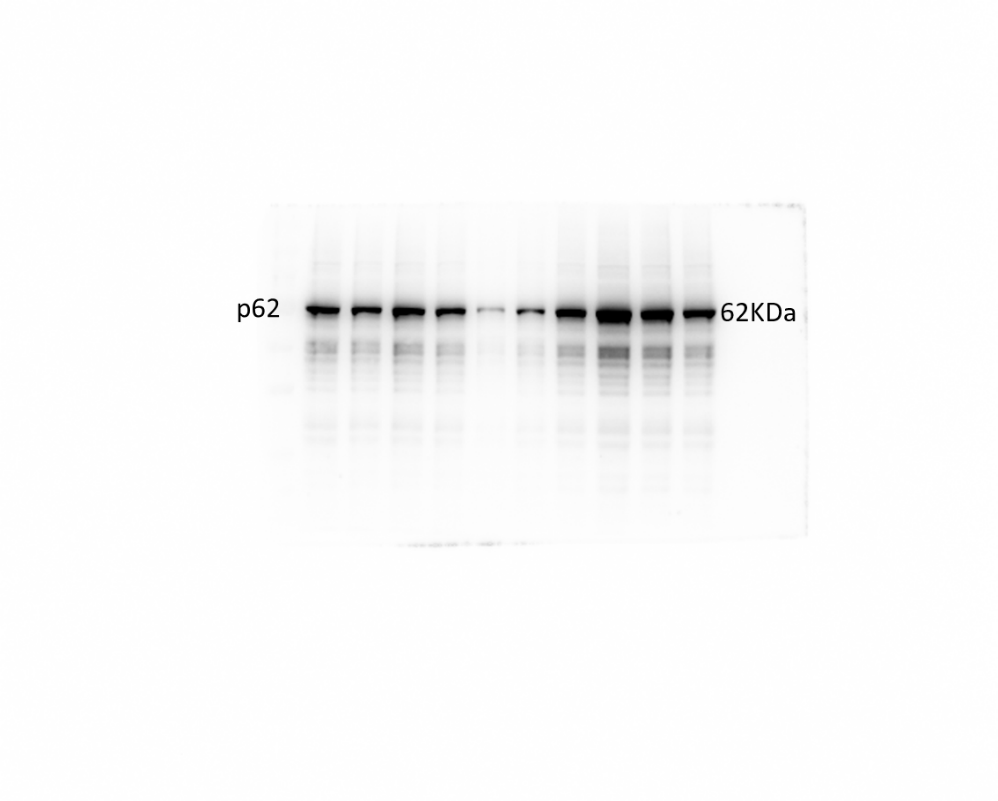
**

**
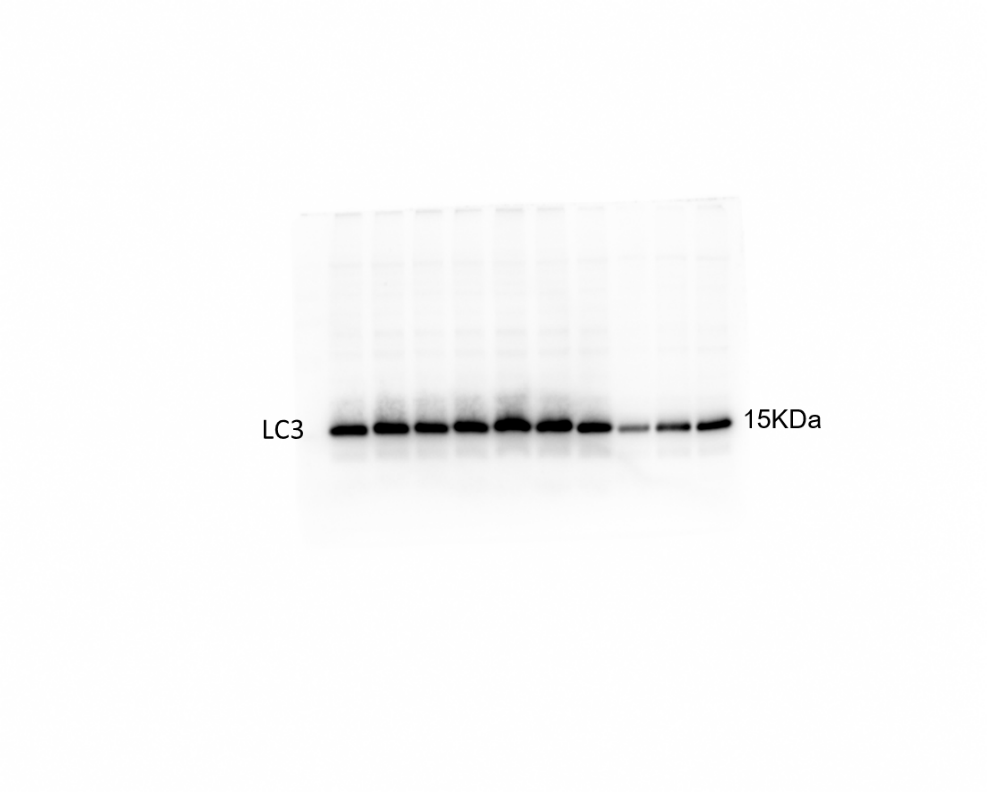

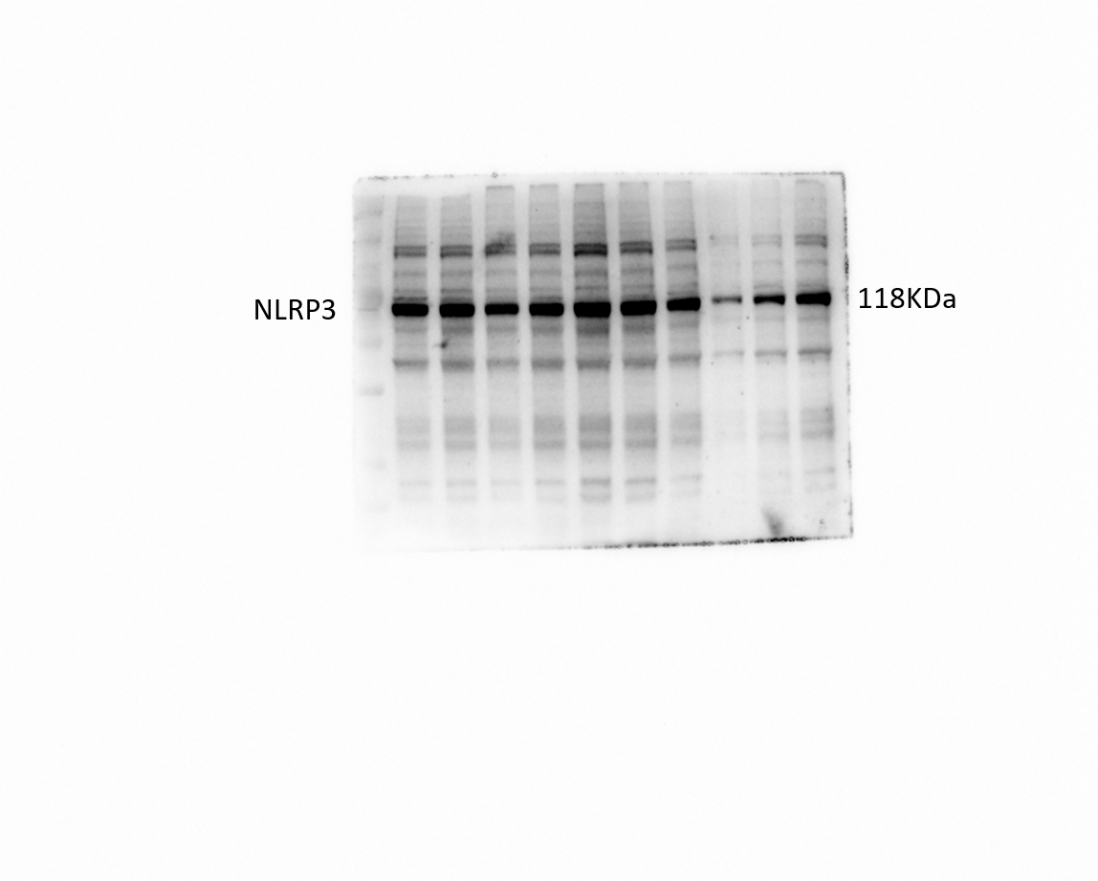

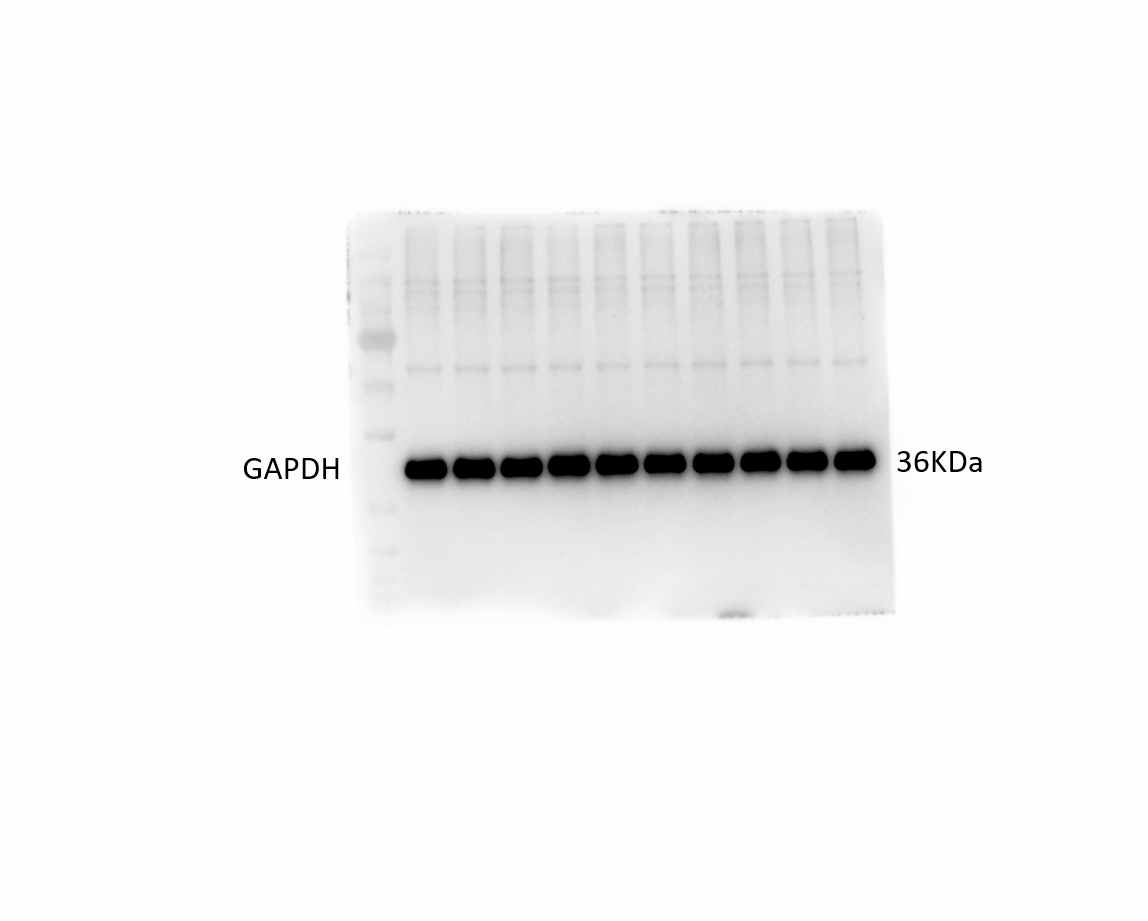
**

**Figure 6F**

**Grouping from left to right: Normal, TAC, TAC+INF39, TAC+MiR-26a-5p mimic NC, TAC+MiR-26a-5p mimic, TAC+MiR-26a-5p mimic+3-MA, TAC+MiR-26a-5p inhibitor NC, TAC+MiR-26a-5p inhibitor, TAC+MiR-26a-5p inhibtor+Rapamy**

**
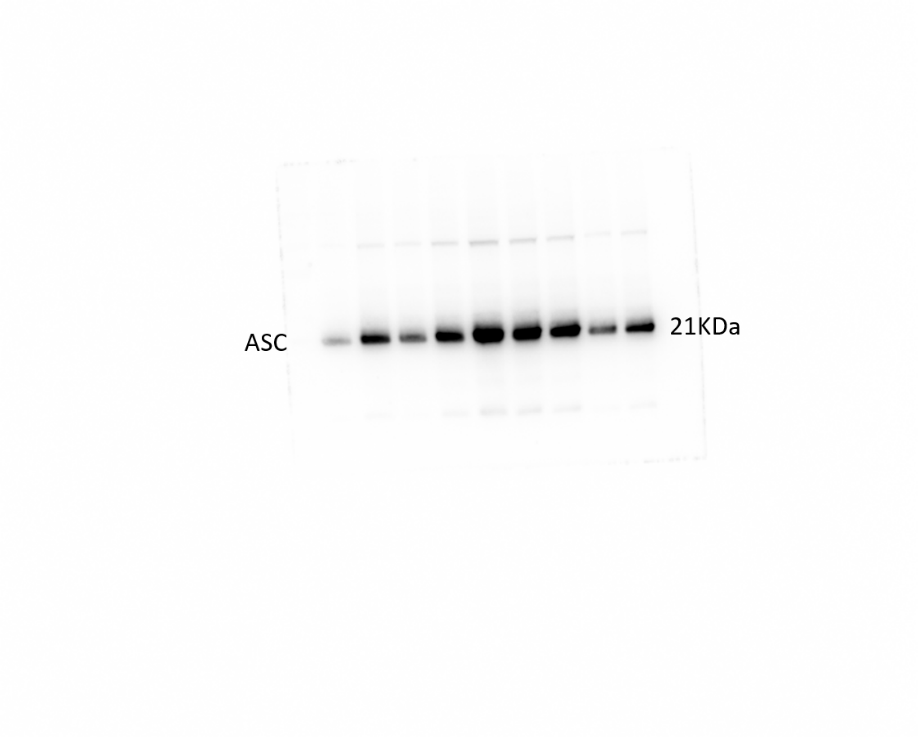

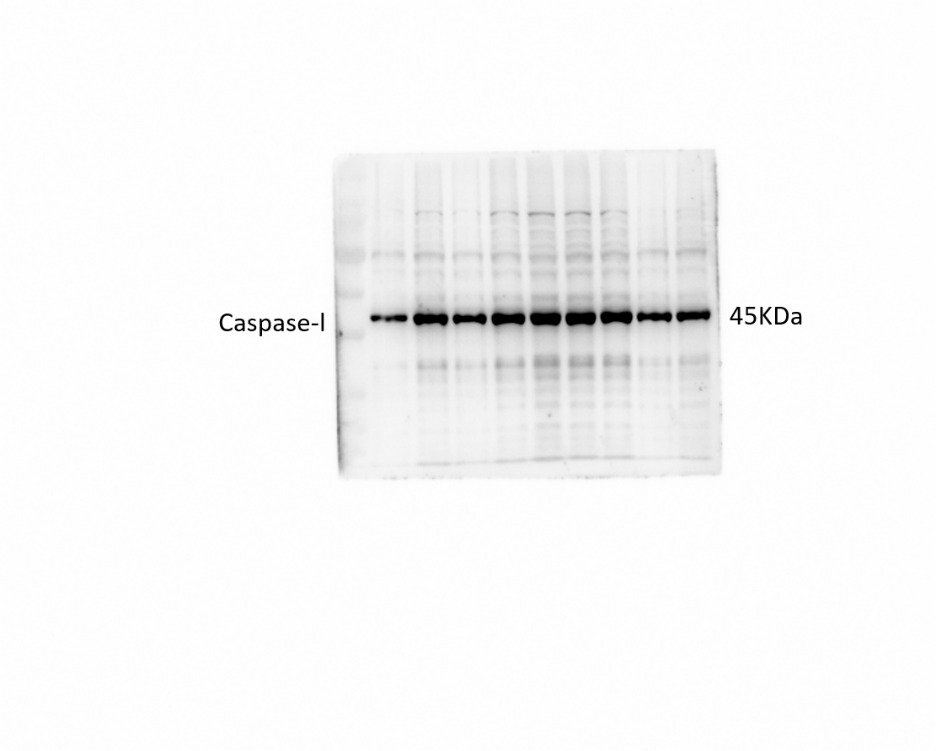
**

**
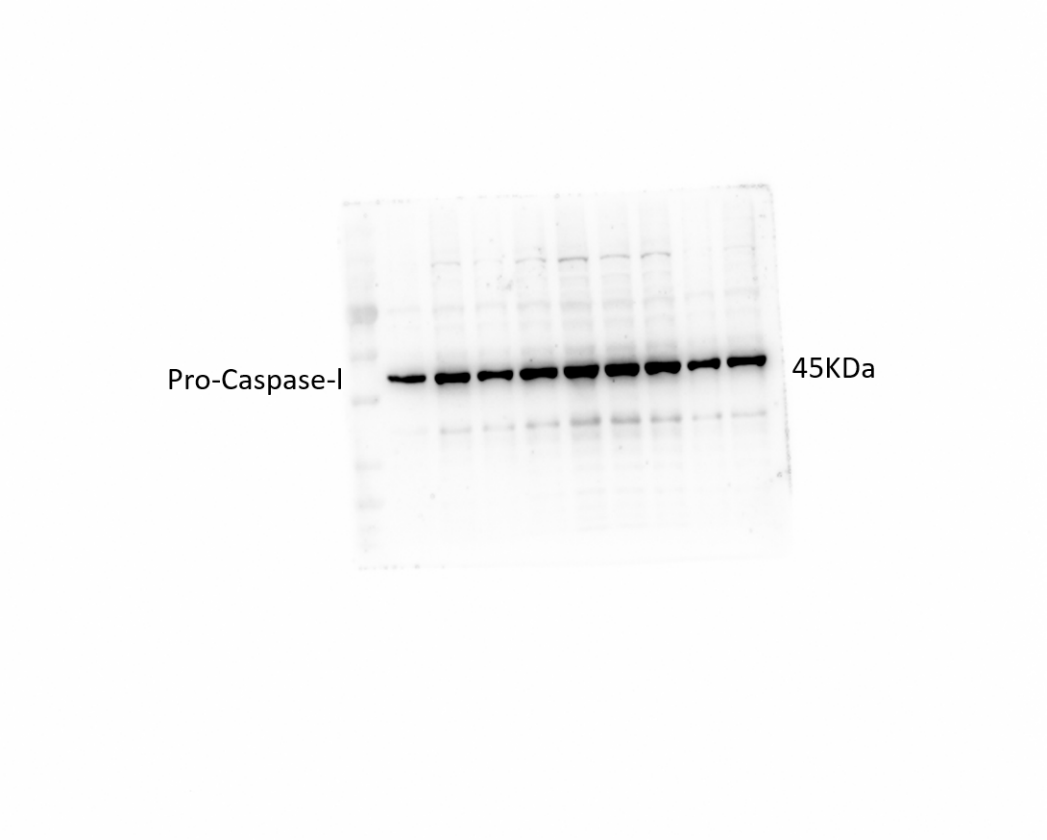

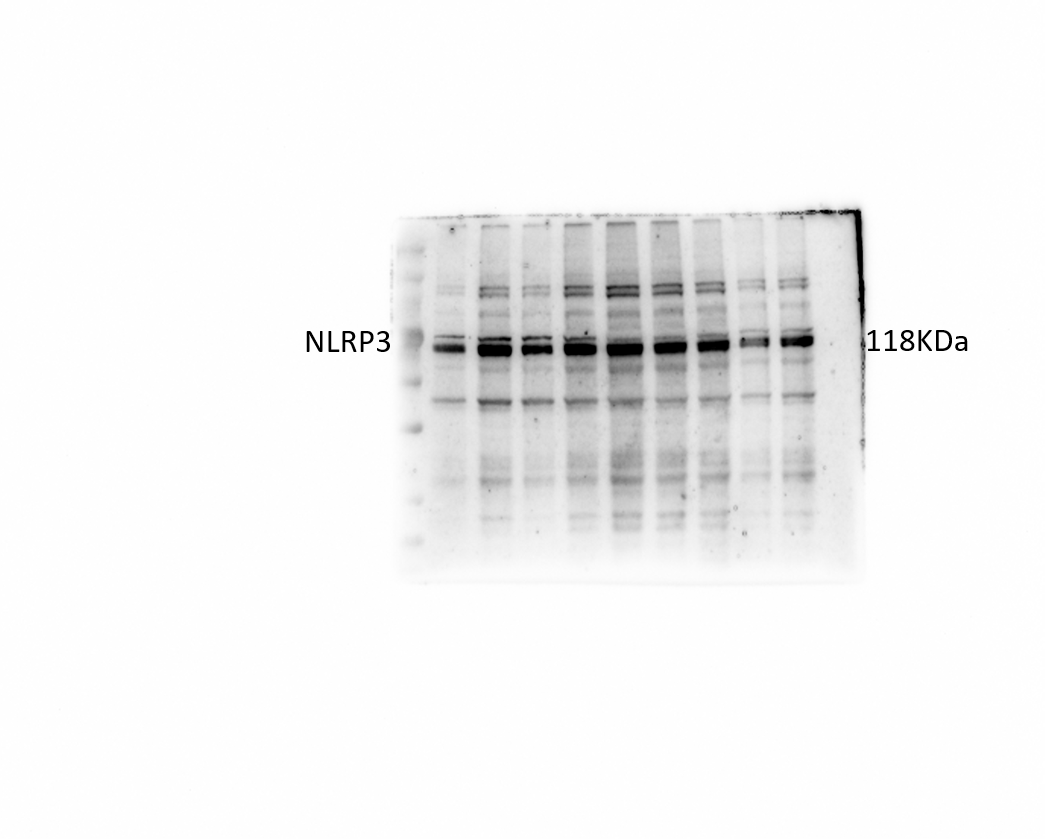

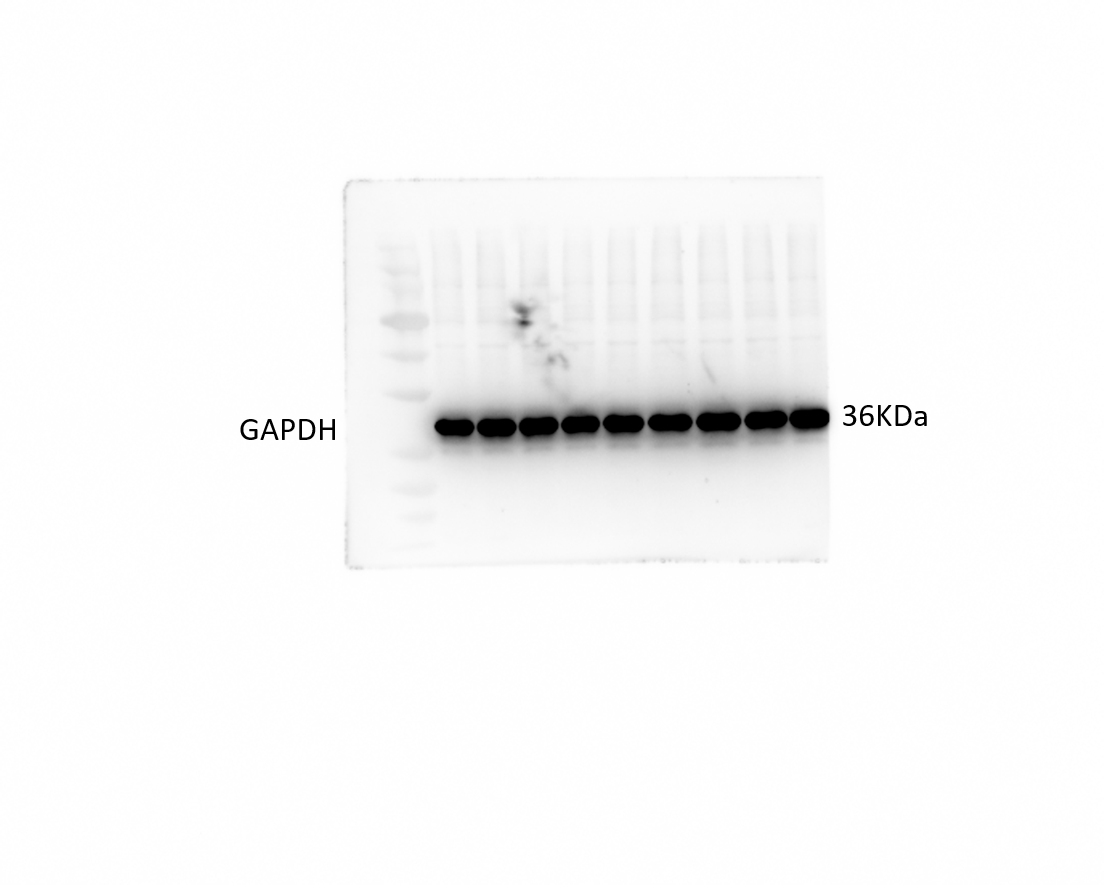
**
